# Does classroom-based crew resource management training have an effect on attitudes between doctors and nurses?

**DOI:** 10.5116/ijme.56f5.6804

**Published:** 2016-04-09

**Authors:** Christina K.W. Chan, Hang-kwong So, Wing-yiu Ng, Pei-kei Chan, Wei-ling Ma, Kin-ling Chan, Siu-ha Leung, Lap-Yin Ho

**Affiliations:** 1 Multidisciplinary Simulation and Skills Centre, Queen Elizabeth Hospital, Hong Kong; 2 Department of Quality and Safety, Queen Elizabeth Hospital, Hong Kong; 3 Department of Obstetrics and Gynaecology, Queen Elizabeth Hospital, Hong Kong; 4 Accident and Emergency Department, Queen Elizabeth Hospital, Hong Kong

**Keywords:** Patient safety, crew resource management, CRM, healthcare, teamwork

## Abstract

**Objectives:**

To evaluate participant reactions and attitudes to crew resource management teamwork classroom-based training by comparing Likert responses before and after the intervention and exploring potential differences in attitudes across the different healthcare professionals.

**Methods:**

Between 26 January and 27 March, 2015, a randomly selected sample of 240 frontline healthcare professionals offering direct patient care were recruited to undergo a 4-hour crew resource management classroom-based training programme. Participants were asked to complete a 22-item human factors attitude survey before and after training and a 10-item end-of-programme evaluation. Paired samples t-test was used to assess differences between the participants' pretest and posttest scores on each item.

**Results:**

A total of 167 (70%) from 17 different specialties underwent the training and 164 (68.3%) completed (139 nurses, 25 doctors) the survey. The nurses were of similar age to the doctors (38.2 vs 36.9, p=0.83) and were more likely to be women (75.6% vs 24.6%, p <0.001). Human factors attitude survey findings indicated that nurses valued the experience highly compared to doctors. The responses among the nurses revealed significant attitude shifts (p <0.05) in 20 of the 22 items whereas this was the case only for 9 items among the doctors.

**Conclusions:**

Overall, the crew resource management classroom-based training programme appeared to have a positive effect on frontline healthcare professionals’ attitudes. The implementation of such programme is feasible and acceptable, especially for nurses, in a public hospital setting in Hong Kong.

## Introduction

Effective teamwork is found to play an important role in the healthcare industry and many underlying causes of adverse events in healthcare are due to the absence of non-technical skills, such as teamwork and communication, rather than technical skills.^1^^,^^2^ This has been found and well recognized in the aviation industry. Therefore, specialized training programmes, such as Crew Resources Management (CRM), was developed to improve safety behaviour and minimize human error related to air transport accidents.^3^   Since it has high face validity, many healthcare organizations have adopted these principles and applied them to their training to help improve patient safety. Some of the key areas that CRM emphasizes are communication, decision-making, teamwork, situational awareness, leadership and performance feedback.^4^

CRM training has become a common teaching method for healthcare organizations. A recent study found that classroom-based training in hospitals resulted in improvement of knowledge (mean difference=1.5, p=0.002) and teamwork behaviour (mean difference=2.69, p=0.027).^5^  Other studies have also found that CRM training improves clinical team performance^6^ and patient outcomes (e.g. a decrease in mortality).^7^^,^^8^ In relation to healthcare, the value of CRM has been demonstrated in various clinical departments, such as intensive care units (ICU),^9^ operating theatres,^10^ paediatric,^11^ and emergency departments.^12^  

The Multidisciplinary Simulation and Skills Centre in Queen Elizabeth Hospital (QEH) was set up in 2011. The training centre has built quality assurance (QA) from the beginning. The first step in QA was to ensure that our curriculum fulfils the CRM training guideline. This was achieved through accreditation in March 2014. As educators in the healthcare profession, we need to understand the nurses and doctors’ reactions toward the training, the shift in attitudes before and after the training and the differences in attitudes between doctors and nurses.      

## Methods

### Study participants

We randomly selected 240 healthcare professionals from QEH who worked as a team and provided direct care for the patients. Only those who were associate consultants, resident specialists, residents, advanced practice nurses and registered nurses were invited for this study. Participants were informed by the department heads about the training and participation was voluntary. A reminder email was sent to all participants one week before the study and informed consent was obtained on the day of the study.

Of the 240 participants who were invited to join the study, 164 of them showed up for the training (139 nurses and 25 doctors). The mean ages of nurses and doctors were 38.2 years and 36.9 years, respectively. Over 60% of them had more than 10 years of work experience and 24% of them had heard of CRM. Amongst the 164 participants, 42 were from the department of Medicine (25.6%), 8 (4.9%) were from the department of Surgery, 13 (7.9%) were from the department of Obstetrics & Gynaecology, 16 (9.7) were from the department of Paediatrics, 6 (3.7%) were from the Accident & Emergency department, 8 (4.9%) were from the Intensive Care Unit, 22 (13.4%) were from the department of Anaesthesiology & Operating Theatre Services, 10 (6.1%) were from the department of Clinical Oncology, 9 (5.5%) were from the department of Orthopaedics and Traumatology, 9 (5.5%) were from the department of Radiology & Imaging, and 21 (12.8%) from other departments such as Private Clinic and Neurosurgery.

### CRM classroom-based training development

Between 26 January and 27 March, 2015, MDSSC conducted 12 CRM classroom-based training programmes. The programmes were supported by the Hong Kong Hospital Authority (HA) and their goal was to improve patient safety. The programme curriculum design, content and training objectives addressed the staff learning needs among the four high-risk departments: Obstetrics & Gynaecology, Anaesthesiology & Operating Theatre Services, Intensive Care Unit, and Accidental & Emergency.^13^ The training was a 5-hour programme led by two CRM certified instructors and emphasized leadership, communication, assertiveness, and situational awareness. Each session began with a brief introduction, followed by various games, video clips, discussion and exercises.

### Leadership

Leadership refers to the qualities that promote good teamwork, the ability to inspire team members to work coordinately, and the capability to give direct commands and listen to team members’ opinions. The training emphasizes interpersonal skills and the importance of leaders as well as followers to mutually respect each other to pursue common goals.

### Communication

Communication is more than just talking. It is the process of using words and actions to convey meaning. “Closed loop” communication and “Situation-Background-Assessment-Recommendation (SBAR)” are effective tools for team communication and this session introduced how to use them effectively. Participants had the opportunity to learn and practice the difference between using open loop versus closed loop communication and non-SBAR versus SBAR. 

### Assertiveness

Assertiveness is an important communication skill that involves standing up for one’s rights while respecting the rights of others. The purpose of this session is to teach the appropriate use of assertiveness to speak out when patient safety is at risk. The “5-Step Assertion Model”^14^ is a guide that can help to improve assertiveness in the interest of patient safety. The steps involve: (1) getting a person’s attention; (2) expressing concern; (3) stating the problem; (4) proposing action; and (5) reaching a decision. In the training, participants are taught to use critical language derived from the CUS model - “I am concerned”, “I feel uncomfortable” and “it is not safe”.^15^

### Situational awareness

Situational awareness refers to the ability to recognize adverse events and identify red flags. This session focuses on training the participants on how to manage red flags by performing the following three steps - See it, Say it and Fix it. Once the threats have been identified, open and assertive communication should apply; then team leaders should gather all the information and opinions from the teammates before making the final decision.

In summary, CRM classroom-based training teaches the participants explicit behavioural strategies to strengthen their skills through team building games and open discussions. Training enables participants to focus on the importance of patient safety and to recognize how the skills improve their performance at work. 

**Table 1:** Means and standard deviations for human factor attitude survey (n=164)

Item	Pre-training Mean (SD)	Post-training Mean (SD)	p-value
1. Team leader and team members can improve decision-making skills through training.	3.96 (0.56)	4.23 (0.51)	<0.001
2. Team leader should encourage team members’ to raise questions during normal operations and emergencies.	3.94 (0.61)	4.30 (0.54)	<0.001
3. My performance is not adversely affected by working with an inexperienced or less capable team member.	2.94 (0.84)	3.20 (0.88)	<0.001
4. Team members should question the decisions or actions of the team leader during a procedure.	3.65 (0.63)	4.14 (0.52)	<0.001
5. Prior to the procedure, it is important for all team members to be familiar with the tasks and responsibilities of the other members of the team.	4.09 (0.57)	4.24 (0.53)	0.003
6. My ability to detect adverse situations has a direct relationship to the quality of decisions I make.	3.82 (0.59)	4.14 (0.47)	<0.001
7. It is necessary for the team leader to explicitly tell team members that he/she wants their input.	3.85 (0.57)	4.14 (0.47)	<0.001
8. In response to unplanned events, the team leader should verbalize plans and ensure that the information is understood and acknowledged by all team members.	4.08 (0.61)	4.29 (0.53)	<0.001
9. Good communication and coordination are as important as technical proficiency for the safety of operative procedures.	4.34 (0.57)	4.48 (0.54)	0.009
10. With trained and experienced staff members, good decisions are almost automatic in the planning and executing of operational requirements.	3.82 (0.65)	3.91 (0.68)	0.12
11. A debriefing and critique of procedures and decisions after each event is an important part of developing and maintaining effective team coordination.	3.95 (0.58)	4.15 (0.56)	<0.001
12. A discussion of alternative methods does not make the team leader appear indecisive.	3.55 (0.75)	3.90 (0.61)	<0.001
13. Once team leaders have made a decision and announced it to the team, they should listen to the reservations on their decision from team members.	3.82 (0.59)	3.96 (0.53)	0.01
14. Staff in my own department need training to “speak up” when they see something that is not right.	3.71 (0.75)	4.09 (0.74)	<0.001
15. Supervisors should be able to provide specific instruction and feedback on teamwork skill attainment.	3.99 (0.39)	4.17 (0.45)	<0.001
16. When making decisions, I gather as much information as time allows before making/executing my decision.	3.92 (0.56)	4.13 (0.51)	<0.001
17. It is just as important to note and debrief what was done well as it is to note and debrief what needs improvement.	3.95 (0.53)	4.15 (0.51)	<0.001
18. There are circumstances where another team member should assume control of the event.	3.58 (0.65)	3.82 (0.67)	<0.001
19. Recognizing adverse events is one of the most important keys to overall patient safety.	4.17 (0.59)	4.25 (0.56)	0.12
20. If I perceive a problem with the event, I will speak up, regardless of who might be affected.	3.66 (0.67)	4.07 (0.61)	<0.001
21. The team formation and decision-making skills of team leaders are as important as their technical skills.	3.91 (0.67)	4.18 (0.48)	<0.001
22. During any procedure or shift, and in response to unplanned or unbriefed contingencies, the team leader should verbalize plans for procedures and should be sure that the information is understood and acknowledged by all team members.	4.04 (0.51)	4.20 (0.53)	<0.001

### Data collection

The Human Factors Attitude Survey (HFAS) was used to assess the attitudinal shifts related to team behaviour. This instrument was modified from an aviation-based attitudinal survey developed by the University of Texas and NASA.^16^^,^^17^ The HFAS has a total of 23 items and is characterized by good internal reliability (Cronbach’s Alpha = 0.89). All questions were reviewed by expert doctors and nurses for content and face validity. One question was not used due to inapplicability to the study. Participants were asked to indicate their agreement with each question on a five-point Likert scale, with 1 indicating ‘strongly disagree’ and 5 ‘strongly agree’. The survey was administered before and after the CRM training programme. Participant reactions to the training were measured by post-programme evaluation. The study proposal was approved by the Institutional Review Board of Kowloon Central Cluster Ethics Committee.

### Data analysis

Descriptive data were collected, analyzed and reported as mean±standard deviation. Pre- and post-test HFAS were analyzed by student’s t test. A p-value of less than 0.05 was considered significant. All analyses were performed using STATA 13 (College Station, Texas, USA).

## Results

Responses to the HFAS are summarized in Table 1. Significant improvements (p<0.05) after the intervention were found in 20 of the survey items. The items related to team leader were viewed as significantly improved at decision-making skills, encouraging team members to raise questions, explicitly telling team members that he/she wants their input, verbalizing plans, and providing specific instruction and feedback on teamwork skill attainment (p<0.001). Participant responses to the ability to detect adverse situations, the importance of debriefing, “speaking up”, and decision-making, and to control the event by team members were shown to have significantly improved after the training (p<0.001). There was no change in the attitude of good decisions are almost automatic with trained and experienced staff members and recognizing adverse events is the most important keys to overall patient safety (p=0.12). Cronbach’s alpha values for HFAS were found to be 0.84 and 0.90 at pre- and post-intervention, respectively, consistent with previous HFAS.^4^^,^^18^  

**Table 2:** Differences in attitudes as measured by the human factor attitude survey between nurses and doctors (n=164)

Item No.	Nurse (n= 139)				Doctor (n=25)		
	Pre Mean (SD)	Post Mean (SD)	p-value		Pre Mean (SD)	Post Mean (SD)	p-value
1	3.96 (0.57)	4.21 (0.52)	<0.001		3.88 (0.53)	4.36 (0.49)	<0.001
2	3.91 (0.59)	4.31 (0.54)	<0.001		4.12 (0.73)	4.28 (0.54)	0.33
3	3.04 (0.82)	3.27 (0.87)	0.003		2.40 (0.76)	2.84 (0.85)	0.02
4	3.69 (0.61)	4.16 (0.51)	<0.001		3.44 (0.71)	4.04 (0.54)	<0.001
5	4.05 (0.56)	4.24 (0.52)	<0.001		4.32 (0.63)	4.20 (0.58)	0.38
6	3.78 (0.61)	4.12 (0.47)	<0.001		4.00 (0.41)	4.24 (0.36)	0.03
7	3.85 (0.56)	4.12 (0.46)	<0.001		3.88 (0.60)	4.24 (0.52)	0.009
8	4.06 (0.59)	4.28 (0.54)	<0.001		4.20 (0.71)	4.32 (0.48)	0.38
9	4.33 (0.58)	4.49 (0.54)	0.008		4.36 (0.49)	4.40 (0.50)	0.75
10	3.82 (0.65)	3.94 (0.70)	0.06		3.80 (0.65)	3.72 (0.54)	0.54
11	3.96 (0.58)	4.15 (0.55)	<0.001		3.92 (0.64)	4.16 (0.62)	0.06
12	3.50 (0.73)	3.86 (0.64)	<0.001		3.80 (0.87)	4.08 (0.40)	0.13
13	3.81 (0.61)	3.96 (0.54)	0.02		3.88 (0.44)	3.96 (0.45)	0.43
14	3.72 (0.76)	4.08 (0.74)	<0.001		3.64 (0.70)	4.16 (0.75)	0.007
15	3.99 (0.42)	4.16 (0.45)	<0.001		4.04 (0.20)	4.24 (0.44)	0.02
16	3.89 (0.57)	4.12 (0.51)	<0.001		4.08 (0.49)	4.24 (0.52)	0.21
17	3.91 (0.54)	4.12 (0.49)	<0.001		4.12 (0.44)	4.32 (0.63)	0.17
18	3.59 (0.64)	3.79 (0.68)	<0.001		3.64 (0.70)	4.00 (0.65)	0.03
19	4.12 (0.58)	4.21 (0.56)	0.13		4.44 (0.58)	4.48 (0.51)	0.71
20	3.71 (0.64)	4.05 (0.61)	<0.001		3.44 (0.77)	4.20 (0.65)	<0.001
21	3.91 (0.67)	4.16 (0.49)	<0.001		3.96 (0.68)	4.28 (0.46)	0.07
22	4.04 (0.53)	4.19 (0.54)	0.003		4.08 (0.40)	4.24 (0.52)	0.10

The differences in attitudes between nurses and doctors are outlined in Table 2. Except for 2 items, HFAS responses among the nurses revealed a significant attitude shift (p<0.05) after CRM classroom-based training. Only questions 10 and 19 failed to produce statistically significant differences in pre- and post-test responses. The highest mean pretest score on the HFAS was 4.33 for question 9 and it still remained the highest in the post-test with a score of 4.49 (p=0.008). On the other hand, HFAS analysis revealed significant differences between the pre- and post-test mean score in 9 of 22 items among the doctors. For question 19, the mean pre-test score was 4.44 which was found to be the highest score on the HFAS and did not improve much after training.

### Evaluation of the programme

In general, participants found that the CRM classroom-based training programme was useful, relevant and interesting. A summary of participant ratings for the full 10 questions is provided in Table 3.

When asked to list the learning points applicable to work, participants most commonly responded to the CRM elements, such as situational awareness, leadership, assertiveness and communication skills.

## Discussion

A key purpose of CRM training is to overcome the fallibility of human beings, by implementing non-technical skills such as communication, leadership, situational awareness and assertiveness in a high-risk setting.^19^^-^^21^ We developed, implemented and evaluated a training programme aimed at improving attitudes towards patient safety among frontline healthcare professionals. In developing the CRM classroom-based training, we worked closely with various doctors working in an acute setting and focused intensively on the four most important CRM elements that we mentioned previously. For example, participants were taught to use the SBAR method for enhancing communication and to address the complete and concise transfer of information between healthcare professionals.

**Table 3:** Participant responses regarding the CRM classroom teaching (n=164)

Question from Programme Evaluation	Mean ± SD (Responses %)
How interesting did you find this lecture?	3.98±0.63 (83.53)
How useful did you find this lecture?	4.02±0.61 (85.97)
What did you think about the structure of the teaching?	3.98±0.59 (84.75)
Did you think that the case studies were useful and relevant?	4.01±0.63 (83.53)
Did you think that the discussion was useful?	3.98±0.64 (81.70)
What did you think of the instructor in terms of teaching?	4.09±0.54 (89.63)
What did you think about the timing of this lecture?	3.84±0.69 (73.78)
What did you think of the relevance of this topic to your job?	4.06±0.68 (84.67)
Did you think that the lecture could increase patient safety and quality care?	4.06±0.65 (84.76)
Overall, how satisfying did you find this lecture?	4.01±0.62 (85.97)

Effective communication plays an important role in improving patient safety. Recent studies support that standardization of communication can improve patient outcomes.^22^^-^^25^ Cornell P et al.^25^ found that SBAR is not just a shortcut for communication but also improves situational awareness, comprehensiveness and clarity especially in a multidisciplinary setting. In addition, the consistency and repeatability of using SBAR benefit staff development and patient outcomes.

A high degree of learning satisfaction shows that frontline healthcare professionals enjoyed learning CRM. From 74 to 89% of participants rated the programme as very high for programme satisfaction, programme usefulness, the relevance to practice, and instructor performance, and the programme could improve patient safety and quality of care. They also indicated the importance of acquiring CRM skills, such as communication skills, assertiveness, decision-making and situational awareness. According to West et al.^26^ CRM principles can be used to improve efficiency, patient safety and staff morale in a healthcare setting. In our study, we found that 20 of 22 questions from HFAS showed statistically significant improvement in attitude towards patient safety and teamwork after the training. Questions 4, 14 and 20 showed the greatest improvement in attitude in which they perceived “speaking up” as important for patient safety. These results are consistent with previous studies.^4^^,^^18^

Our study also demonstrated that the nurses showed a greater positive shift in attitudes towards patient safety than doctors. Although the doctors rated higher overall in pre-tests, the nurses demonstrated a greater mean difference in ratings after training, indicating that doctors may be less receptive to learning CRM through classroom-based training. One reason could be that they emphasize the importance of their roles in patient safety so they easily understand the needs of CRM through games, videos and lectures. Another reason could be that doctors are more interested in scenario-based simulation training rather than classroom-based training. An earlier study^27^ found that emergency medicine residents prefer a high-fidelity environment and it suggested that this might be the most appropriate method for future CRM training.

Although many different teaching programmes in medical education have been using high-fidelity simulation, it is very costly. We believe our programme to be beneficial to our frontline healthcare professionals since it is both inexpensive and logistically feasible. Our programme costs approximately HK$47,500 (US$6,129) for 164 participants, CRM instructors, materials and audio visual support.

There were several limitations to this study. First, the study included a risk of selection bias, as the participants were volunteers. However, the random selection process and the relatively large sample aimed to minimize selection bias. Furthermore, it was undertaken in a single hospital so its generalization to other CRM classroom-based programmes may be limited. Thirdly, the study findings are limited by the number of participants and the changes in attitude were self-reported. Finally, although statistically significant improvements in attitude were shown in the survey, it is not possible to determine whether these improvements are clinically relevant.

## Conclusion

In summary, CRM classroom-based training appears to be highly valued by participants, especially nurses. It focuses on improving interprofessional cooperation and team performance and, ultimately, improves patient safety. Future research should investigate the benefits and impact of CRM training over a longer period of time.

### Acknowledgements

The MDSSC team would like to express its gratitude to Hospital Authority Head Office (HAHO) for their support and contribution in this study. We also thank to the CRM trainers: Dr Chan Wai-man, Dr Kwok Kam-hung, Dr Lam Kam-wah, Dr Mark Hor-kee, Dr Sin Kai-cheuk, Ms Lam Sui-yi, Ms Lee Lai-ha, Ms Lee Wing-sze, Ms Leung Lai-chu and Mr Wong Chi-yip. Without their support the study could have not been completed.

### Conflict of Interest

The authors declare that they have no conflict of interest.
